# Supplementary material for: Studying RNA–DNA interactome by Red-C identifies noncoding RNAs associated with various chromatin types and reveals transcription dynamics
Source: Nucleic Acids Res. 2020 Jun 1;48(12):6699–714. doi: 10.1093/nar/gkaa457 (PMC7337940; doi:10.1093/nar/gkaa457)
Supplement: gkaa457_Supplemental_Files [file gkaa457_supplemental_files.zip › Supplementary Figure and Table Legends.docx]

**Supplementary Figure and Table Legends**

**Figure S1. Development of Red-C and control experiments.** (**A**) Main steps of Red-C procedure. (**B**) Electrophoresis of PCR products from main and control experiments with K562 cells. Expected sizes of PCR products: chimera without both DNA and RNA parts, 163 bp; chimera with DNA part without RNA part, 181 bp; chimera with RNA part without DNA part, > 163 bp; chimera with both DNA and RNA parts, > 181 bp. Asterisk shows free primer. Double asterisk shows primer dimer that is generated in late PCR cycles in the absence of DNA template (the first lane in each four). (**C**) Randomly chosen raw reads (R1) from main and control experiments. The sequences of DNA parts, RNA parts, bridge adapter, and switch template oligo (reverse complement) are highlighted in black, red, gray, and orange colors, respectively. (**D**) Expected sequence of PCR product. Positions of DNA and RNA parts, bridge adapter, and PCR and sequencing primers are indicated. (**E**) Frequency of DNA parts of 18–20 nt and RNA parts of ≥ 14 nt in reads of main and control libraries. The percentage is given of read pairs containing bridge in R1 and GGG in the start of R2 (reads after filter 3, Table S1). (**F**) Length distribution for DNA and RNA parts extracted, respectively, from R1 and R2 reads of the main experiment. Note that the right bar in the right graph includes all RNA parts with lengths higher than the read length (125 nt). (**G**) Number of uniquely mapped DNA and RNA parts in the main library. (**H**) Frequency of mapping of 5’ and 3’ ends of uniquely mapped RNA parts to NlaIII sites. Shuffled control, RNA parts were randomly shuffled within parental chromosomes.

**Figure S2. Flowchart of the Red-C data processing pipeline.** We remove PCR duplicates, trim and filter reads by quality, and identify the bridge and adapter sequences. We extract DNA parts and RNA 3’ parts from the forward reads and RNA 5’ parts from the reverse reads. We map DNA parts of 18–20 bp and RNA 3’ and 5’ parts of ≥14 bp to the reference genome with hisat2 program. We retain only uniquely mapped DNA and RNA parts. We further require that RNA 3’ and 5’ parts be mapped to the same chromosome in a proper orientation at a distance < 10 Kb from each other and filter out the cases when RNA 3’ or 5’ end coincides with NlaIII or MmeI digestion site.

**Figure S3. Identification of RNAs with specific genome distribution patterns.** Each graph shows 10,367 RNAs (all RNA with ≥ 500 contacts) represented as thin gray semitransparent lines drawn in accordance with 4 ranks obtained by sorting RNAs on the basis of ratios between contact frequencies in intervals SM and L (1^st^ rank), L and R (2^nd^ rank), R and T (3^rd^ rank) (see Methods), and finally between contact frequencies in active and repressed chromatin in a specified genomic region (4^th^ rank). The lower the ratio, the lower the rank. RNAs with specific genome distribution patterns are highlighted in separate graphs. Graph in (**A**) shows RNAs with relatively low SM-to-L and high L-to-R and R-to-T ratios that are considered to be enriched in gene-proximal areas. Graph in (**B**) shows RNAs with relatively low SM-to-L and L-to-R and high R-to-T ratios that are considered as XIST-like. Graph in (**C**) shows RNAs with relatively low value of all three ratios that are considered to be distributed throughout the genome. RNAs showing preference for active chromatin are highlighted in red (active-to-repressed ratio > 1, ranked above the mark “1”). RNAs showing preference for repressed chromatin are highlighted in blue (active-to-repressed ratio < 1, ranked below the mark “1”). Specific RNAs or RNA types are further highlighted as indicated to the right of the graphs.

**Figure S4.** **Comparison of RNA–DNA and DNA–DNA contact matrices.** Red-C and Hi-C contact matrices for K562 cells visualized in HiGlass viewer ([https://higlass.io](https://higlass.io/)). Raw Hi-C contacts were obtained from (33).

**Figure S5. Comparison of RNA–DNA interactome between biological replicates of K562 cells.** (**A**) Whole-genome RNA–DNA contact matrices for two biological replicates of K562 cells at a 1 Mb resolution. Pearson correlation coefficients for individual chromosomes are presented to the left. (**B**) RNA–DNA contact matrices for Chr 1 for two biological replicates of K562 cells at a 100 Kb resolution. (**C**) Side-by-side comparison of bottom left halves of RNA–DNA contact matrices shown in (**A**), with the rep1 half being flipped along the diagonal. (**D**) Side-by-side comparison of upper right halves of RNA–DNA contact matrices shown in (**A**), with rep2 half being flipped along the diagonal. (**E, F**) The same as (**C,D**) for RNA–DNA contact matrices shown in (**B**). This flipped view facilitates visual analysis of correlation between RNA–DNA contact matrices. In contrast to DNA–DNA contact matrices of Hi-C, RNA–DNA contact matrices are asymmetric and cannot be compared by simple alignment of the bottom left and upper right halves of the matrices.

**Figure S6. Correlation between the number of contacts for individual RNAs for two biological replicates of K562 cells.** Scatter plot shows the total number of RNA–DNA contacts identified for each RNA in rep1 vs. rep2.

**Figure S7. Comparison of roX1 and roX2 contacts from Red-C with ChAR-seq and GRID-seq.** (**A**) Fold enrichment of roX1 and roX2 on X chromosome relative to average contact frequency at autosomes in Drosophila S2 cells, as determined by Red-C. (**B**) Binding profiles of roX1 and roX2 along X chromosome for Red-C, ChAR-seq, and GRID-seq obtained by aggregating roX1 and roX2 raw contacts (excluding contacts with their own genes) into 20-kb bins. Due to the low coverage of our data compared to ChAR-seq and GRID-seq, we summed contacts of roX1 and roX2 for all datasets, thus obtaining the average binding profile. We used a published ChAR-seq dataset for Drosophila male CME-W1-cl8+ cells (GSE97131) and GRID-seq datasets for S2 cells (GSE82312). We used already processed contacts of ChAR-seq and processed GRID-seq data as follows. RNA and DNA parts were mapped independently to dm3 Drosophila reference genome using hisat2 as described for Red-C. The splice sites were obtained from FlyBase v5.46 gene annotations. Only uniquely mapped RNA–DNA pairs (0–1 mismatch in DNA parts and 0–2 mismatches in RNA parts) were considered for downstream analyses.

**Figure S8. Relative frequency of trans contacts for different regions of mRNAs and protein-coding genes averaged for all chromosomes.** We note that the ratio of cis to trans contacts depends on chromosome length. Indeed, the shorter a chromosome is, the higher the calculated frequency of cis contacts will be due to the prevalence of short distances, as can be seen from Table S7. In Figure 4A we disregard this effect and average frequencies of cis contacts for all chromosomes and frequencies of trans contacts for all chromosomes, followed by presenting obtained values of cis and trans contacts relative to their sum, that is taken to be equal to 1. In the analysis presented here, we first determine frequencies of cis and trans contacts of different regions of mRNAs and protein-coding genes for each chromosome. We obtain six values of frequencies of trans contacts for the following regions: intron regions of protein-coding genes, exon regions of protein-coding genes, exon–intron junctions of mRNA, inner regions of introns of mRNA, inner regions of exons of mRNA, and exon–exon junctions of mRNA. We note that all six values are lower for short chromosomes and higher for long chromosomes. To put six values on the same scale, we normalize each value by the sum of the six values for each chromosome. We then average values for the region of interest over all chromosomes (the values for introns of protein-coding genes are averaged over all chromosomes, the values for exon regions of protein-coding genes are averaged over all chromosomes, and so on). We obtain six average values and present them relative to the maximal value, that is taken to be equal to 1 (the value for mRNA exon–exon junctions). Error bars, SD (*n* = 23, *p*-values are from Tukey’s multiple comparisons test).

**Figure S9. Number of contacts with the genome for exons and introns of mRNAs.** (**A**) Example of gene coverage by RNA parts. Upper track shows the number of RNA parts originating from 100 bp bins across the DYM gene locus. (**B**) Total number of RNA parts mapped to all exons and introns of protein-coding genes. (**C**) Total number of RNA parts mapped to the first three and the last exon–intron pairs. Analysis is done for protein-coding genes with more than four introns. (**D,E**) The same as (**B,C**) after normalization to the total lengths of corresponding exons/introns.

**Figure S10. Contacts of consecutive mRNA segments with the gene body.** (**A**) Contacts of RNA parts originating from exon regions of 12 consecutive gene segments (indicated by vertical red lines) with the gene body and its flanking areas averaged over all mRNAs establishing at least one contact with the gene body or flanking areas (*n* = 11,122). The maximal value of the averaged profile is taken to be equal to 1. Colored area in the background of curves, 95% CI. (**B**) Aggregated contact profile obtained by averaging values of contact profiles shown in (**A**) for bins located in the same position relative to the anchor bin (red line) within the gene body. Error bars, SEM. (**C,D**) The same as (**A,B**) for intron regions.

**Figure S11.** **Intra-gene contacts of mRNAs produced from genes of different length and transcriptional activity.** (**A**) Contacts of first and last exons and introns of mRNA with the body of the encoding gene and its flanking regions averaged over mRNAs produced from long, medium, and short genes. Genes were divided into three groups of equal size (*n* = 3,707) based on length: 845–22,923 bp, short; 22,931–65,272 bp, medium; and 65,294–2,304,638, long. (**B**) The same as (**A**) for genes divided into three groups of equal size (*n* = 3,707) based on the total RNA-seq count: 0–451, weakly expressed; 451–1,701, moderately expressed; and 1,702–148,131, highly expressed. The maximal value among the three profiles is taken to be equal to 1. Colored area in the background of curves, 95% CI.

**Figure S12. Comparison of Red-C and fRIP-Seq data.** (**A**) Intersection of RNAs identified with Red-C with ≥ 1,000 contacts and RNAs showing significant interaction with at least one protein in the fRIP experiment. (**B**) Number of RNAs establishing contacts with indicated proteins among top 1,000 RNAs (1,000+ contacts) with the highest ratio of contact number to RNA-seq signal.

**Table S1. Statistics of read filtering and mapping.**

**Table S2. List of X RNAs with ≥ 100 contacts identified in K562 cells.** Xrna_antisense, newly identified RNAs that intersect a known transcriptional unit and are transcribed in the opposite direction. Coordinates are according to hg19 genome assembly.

**Table S3. List of eRNAs with ≥ 100 contacts identified in K562 cells.** StrEnh_4 and StrEnh_5, strong enhancer RNAs transcribed from chromatin states 4 and 5. WEnh_6 and WEnh_7, weak enhancer RNAs transcribed from chromatin states 6 and 7. Coordinates are according to hg19 genome assembly.

**Table S4. List of RNAs with ≥ 500 contacts identified in K562.** RNAs are sorted according to quantiles obtained in ranking by SM/L, L/R, and R/T ratios (second-to-last column). Column “Active/Repressed ratio” shows the ratio of contact frequencies between active and repressed chromatin in the full genome. RepM, RNAs from UCSC RepeatMasker track. Coordinates are according to hg19 genome assembly.

**Table S5. Proportion of contacts from different regions of mRNA in the total RNA-DNA interactome identified by GRID-seq and Red-C.** We used GRID-seq datasets for human MM.1S cells (GSE82312). RNA and DNA parts were mapped to hg19 genome with hisat2 using the same parameters as for Red-C. Only uniquely mapped RNA–DNA pairs were considered. RNA parts were further annotated as described for Red-C. X RNAs were not annotated.

**Table S6.** **Number of RNAs of a given biotype and total number of contacts for each biotype.** Note that row «all» does not include eRNAs.

**Table S7. Number of cis and trans contacts for different regions of mRNAs (exons, introns, exon–intron junctions, and exon–exon junctions) and protein-coding genes (exons and introns).**
