# Supplementary material for: Studying RNA–DNA interactome by Red-C identifies noncoding RNAs associated with various chromatin types and reveals transcription dynamics
Source: Nucleic Acids Res. 2020 Jun 1;48(12):6699–714. doi: 10.1093/nar/gkaa457 (PMC7337940; doi:10.1093/nar/gkaa457)
Supplement: gkaa457_Supplemental_Files [file gkaa457_supplemental_files.zip › Table_S5.pdf]

	GRID-seq				Red-C			
	MM.1S_rep1		MM.1S_rep2		K562_rep1		K562_rep2	
	No of contacts	%	No of contacts	%	No of contacts	%	No of contacts	%
**all RNAs**	23 561 433	100,00	24 995 021	100,00	18 305 387	100,00	22 536 415	100,00
**mRNA exons**	2 731 862	11,59	2 902 175	11,61	1 338 629	7,31	1 798 857	7,98
**mRNA introns**	17 702 450	75,13	18 773 080	75,11	11 880 779	64,90	14 689 414	65,18
**mRNA exon-intron junctions**	103 564	0,44	109 617	0,44	324 545	1,77	462 744	2,05
**mRNA exon-exon junctions**	16 023	0,07	17 562	0,07	284 329	1,55	485 245	2,15
