# Supplementary material for: Studying RNA–DNA interactome by Red-C identifies noncoding RNAs associated with various chromatin types and reveals transcription dynamics
Source: Nucleic Acids Res. 2020 Jun 1;48(12):6699–714. doi: 10.1093/nar/gkaa457 (PMC7337940; doi:10.1093/nar/gkaa457)
Supplement: gkaa457_Supplemental_Files [file gkaa457_supplemental_files.zip › Table_S6.pdf]

RNA biotype	No of RNAs	No of contacts	No of RNAs with > 100 contacts	No of contacts for RNAs with > 100 contacts
protein coding	16 714	31 814 469	10 904	31 692 638
vlinc	2 112	3 204 789	990	3 181 248
linc	3 653	2 772 365	540	2 733 724
X RNA	1 867	1 314 667	1 867	1 314 667
antisense	3 198	577 263	499	535 002
snRNA	665	497 160	31	492 302
snoRNA	389	273 925	187	270 764
piRNA	30 189	196 016	108	84 675
miRNA	535	26 803	20	20 996
…				
all	66 155	42 156 469	16 048	41 725 299
