# Supplementary figures and images for: Studying RNA–DNA interactome by Red-C identifies noncoding RNAs associated with various chromatin types and reveals transcription dynamics
Source: Nucleic Acids Res. 2020 Jun 1;48(12):6699–714. doi: 10.1093/nar/gkaa457 (PMC7337940; doi:10.1093/nar/gkaa457)

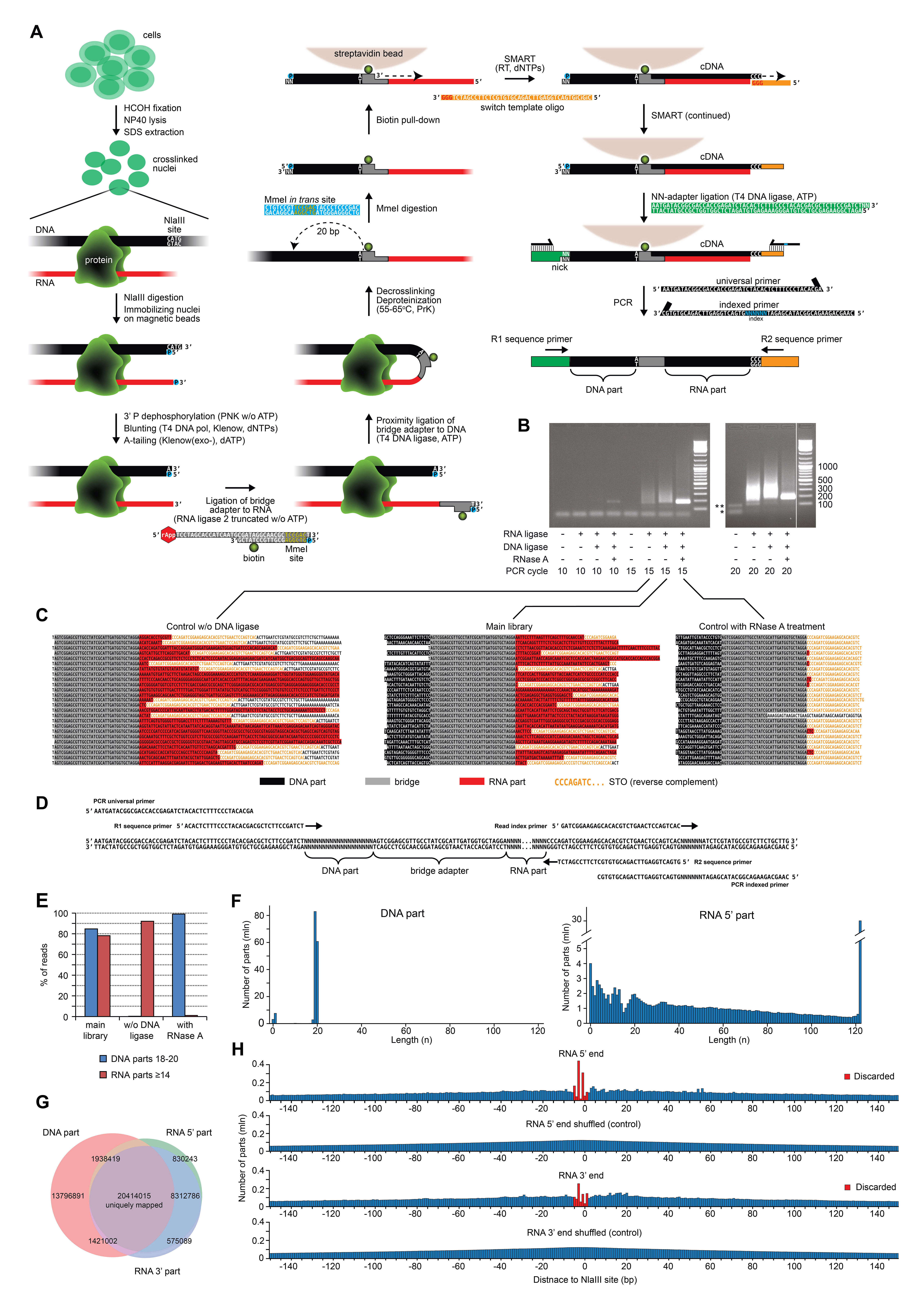

Supplement: gkaa457_Supplemental_Files [file gkaa457_supplemental_files.zip › Fig_S1.jpg]

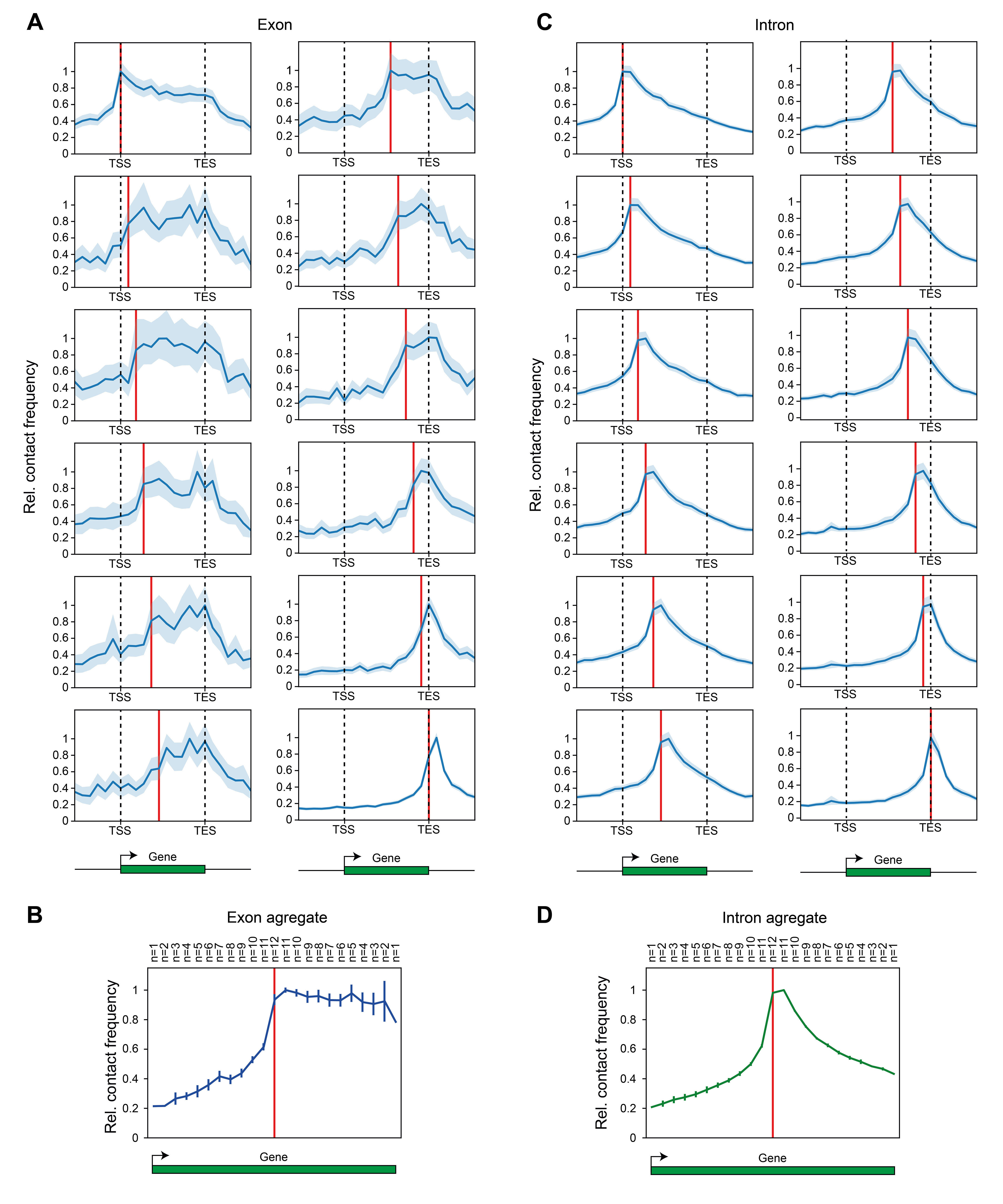

Supplement: gkaa457_Supplemental_Files [file gkaa457_supplemental_files.zip › Fig_S10.jpg]

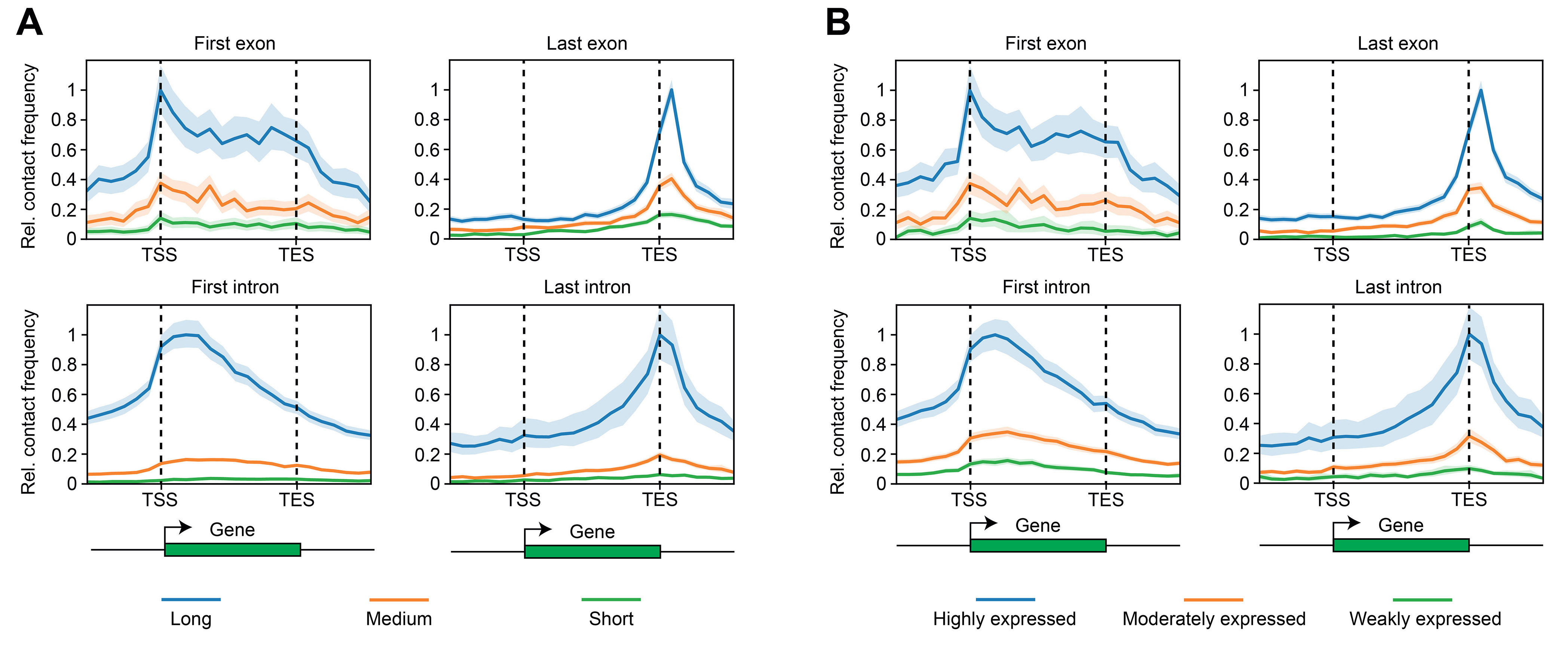

Supplement: gkaa457_Supplemental_Files [file gkaa457_supplemental_files.zip › Fig_S11.jpg]

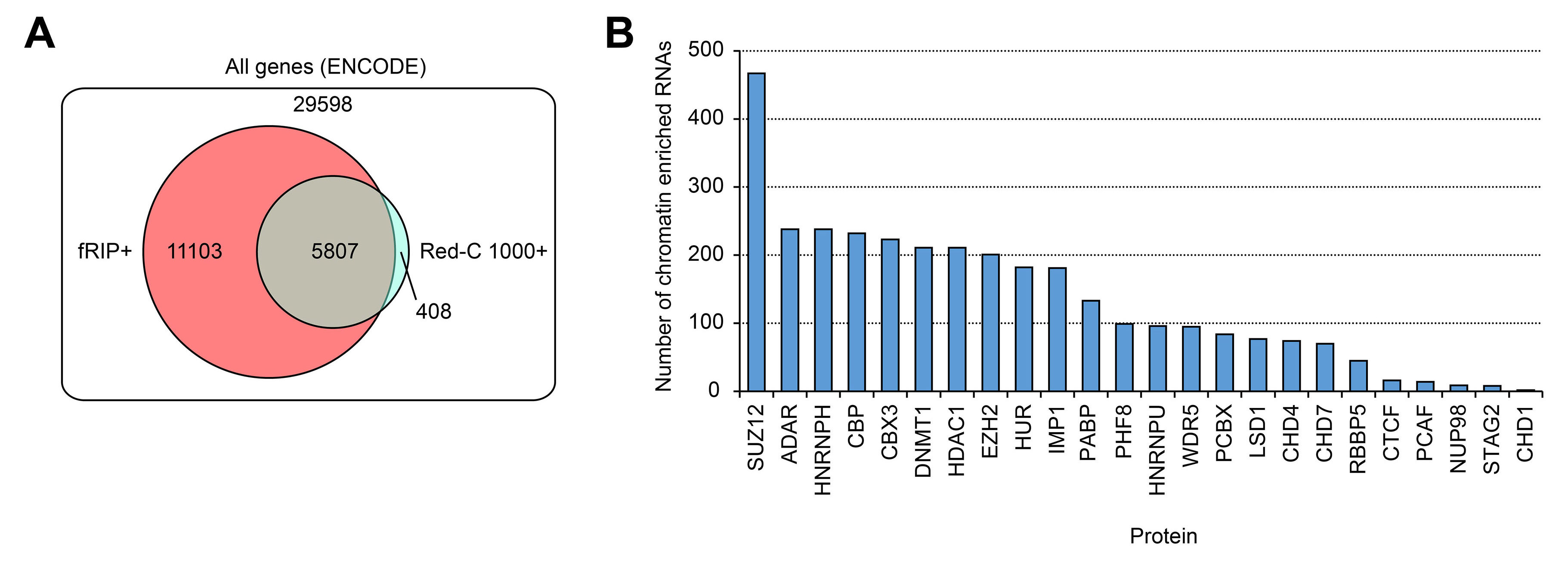

Supplement: gkaa457_Supplemental_Files [file gkaa457_supplemental_files.zip › Fig_S12.jpg]

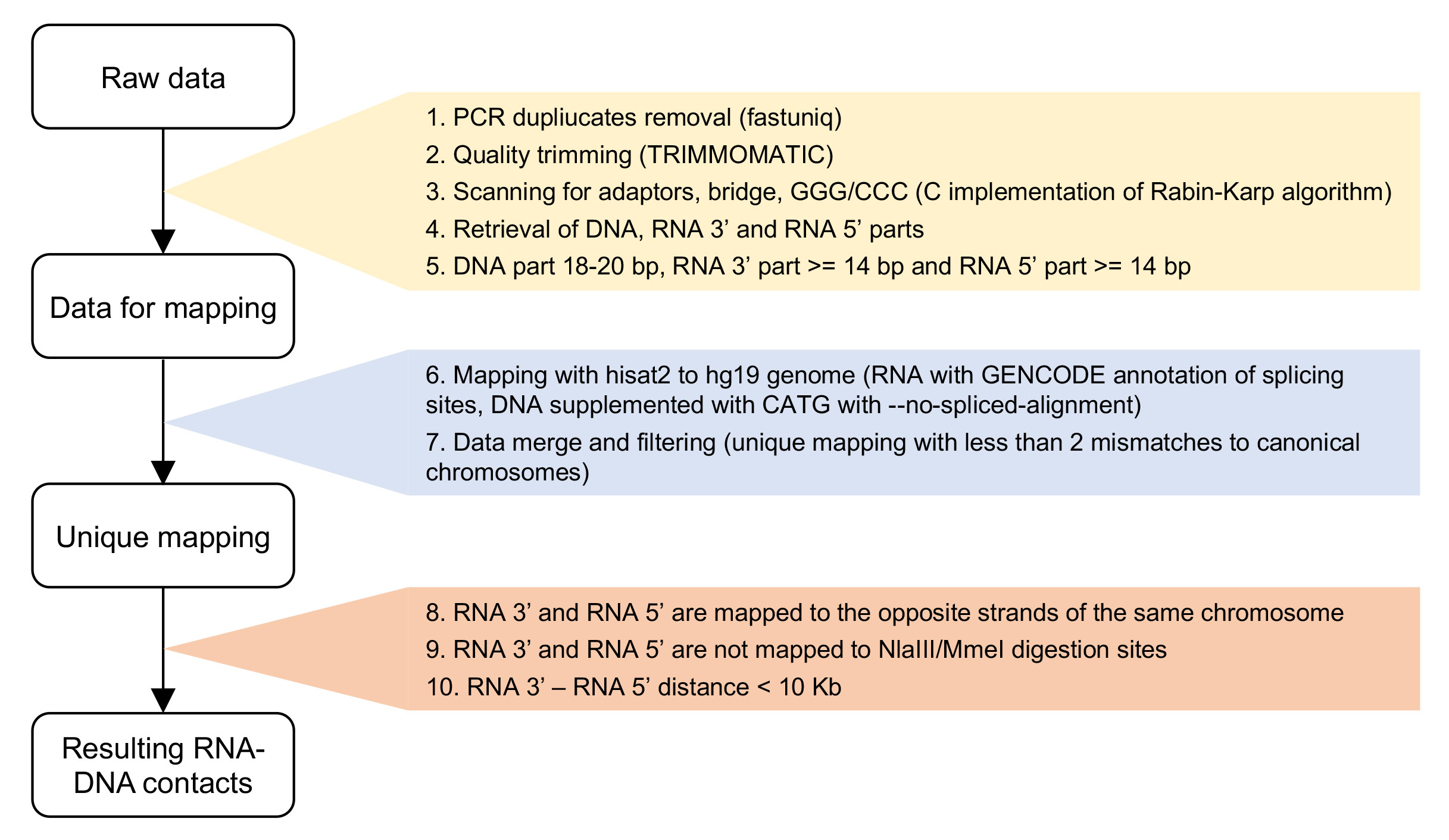

Supplement: gkaa457_Supplemental_Files [file gkaa457_supplemental_files.zip › Fig_S2.jpg]

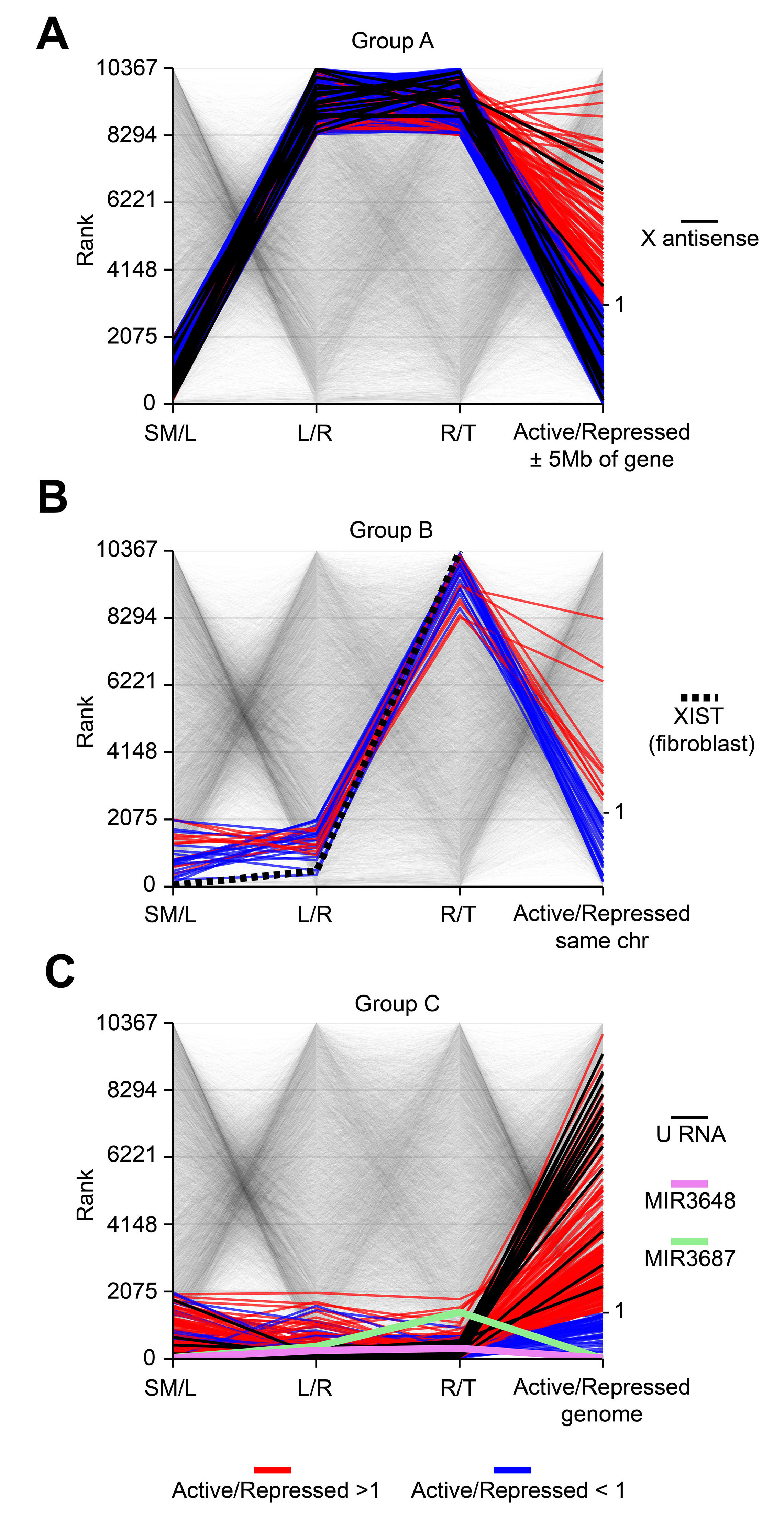

Supplement: gkaa457_Supplemental_Files [file gkaa457_supplemental_files.zip › Fig_S3.jpg]

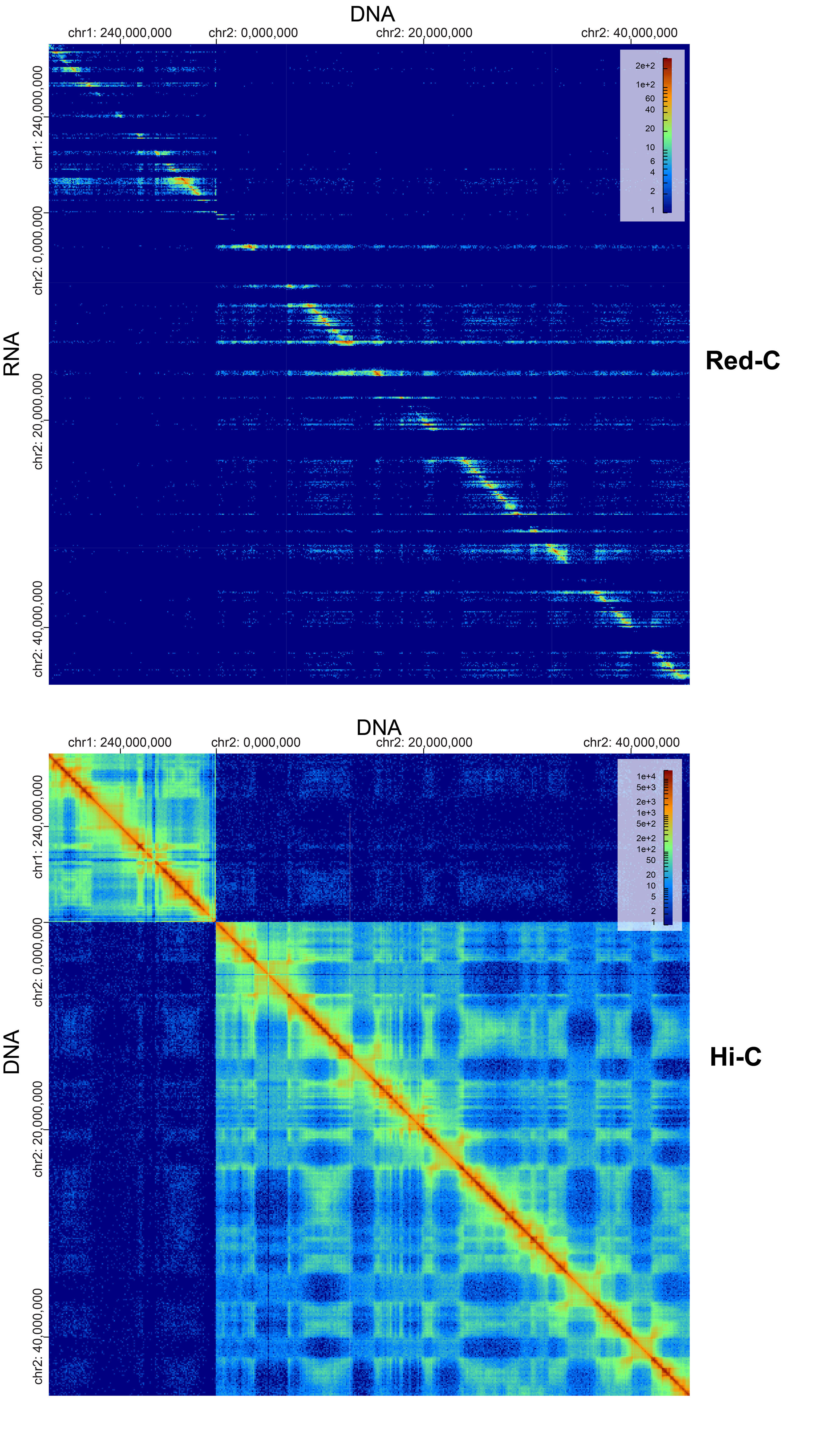

Supplement: gkaa457_Supplemental_Files [file gkaa457_supplemental_files.zip › Fig_S4.jpg]

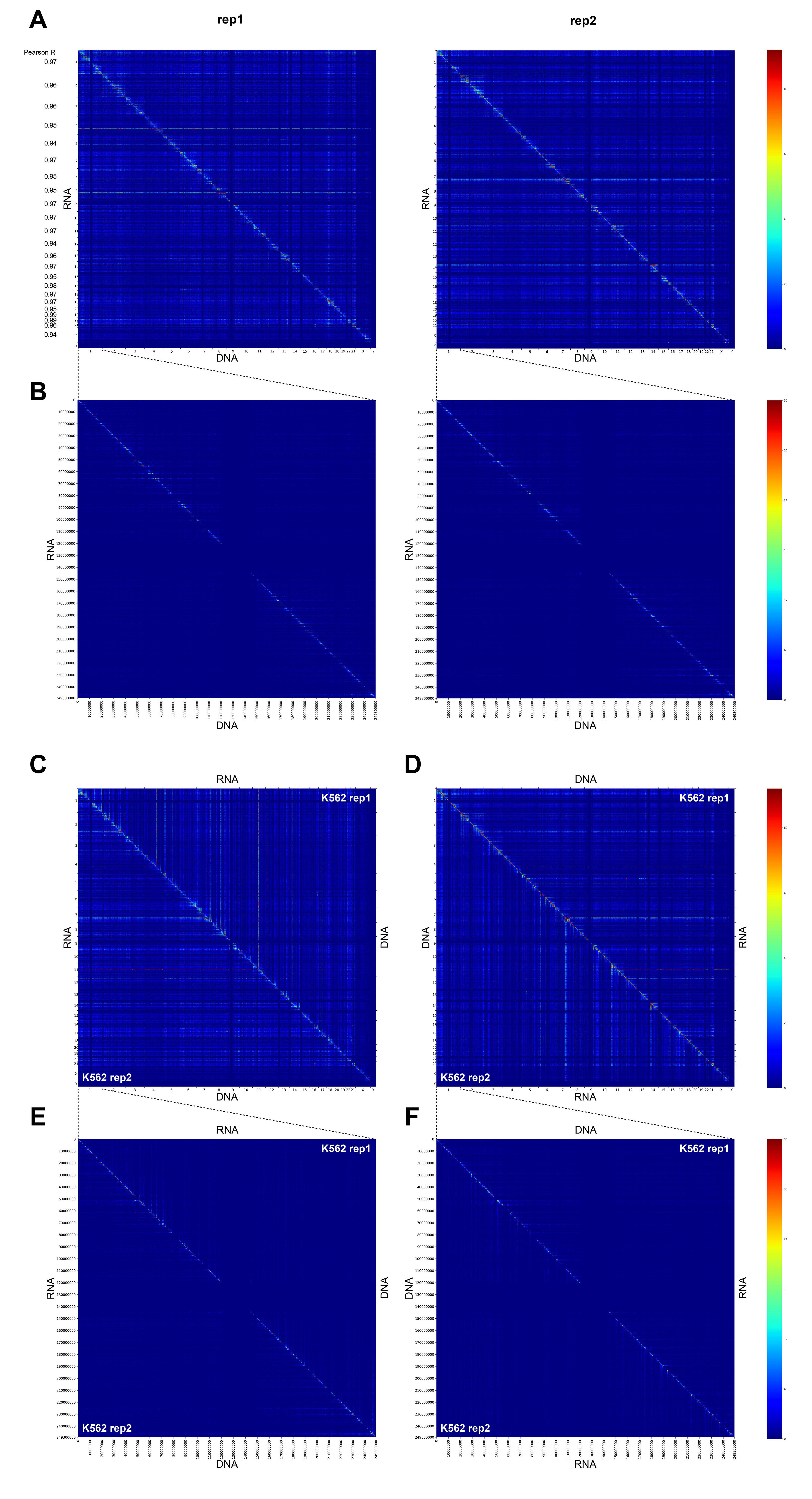

Supplement: gkaa457_Supplemental_Files [file gkaa457_supplemental_files.zip › Fig_S5.jpg]

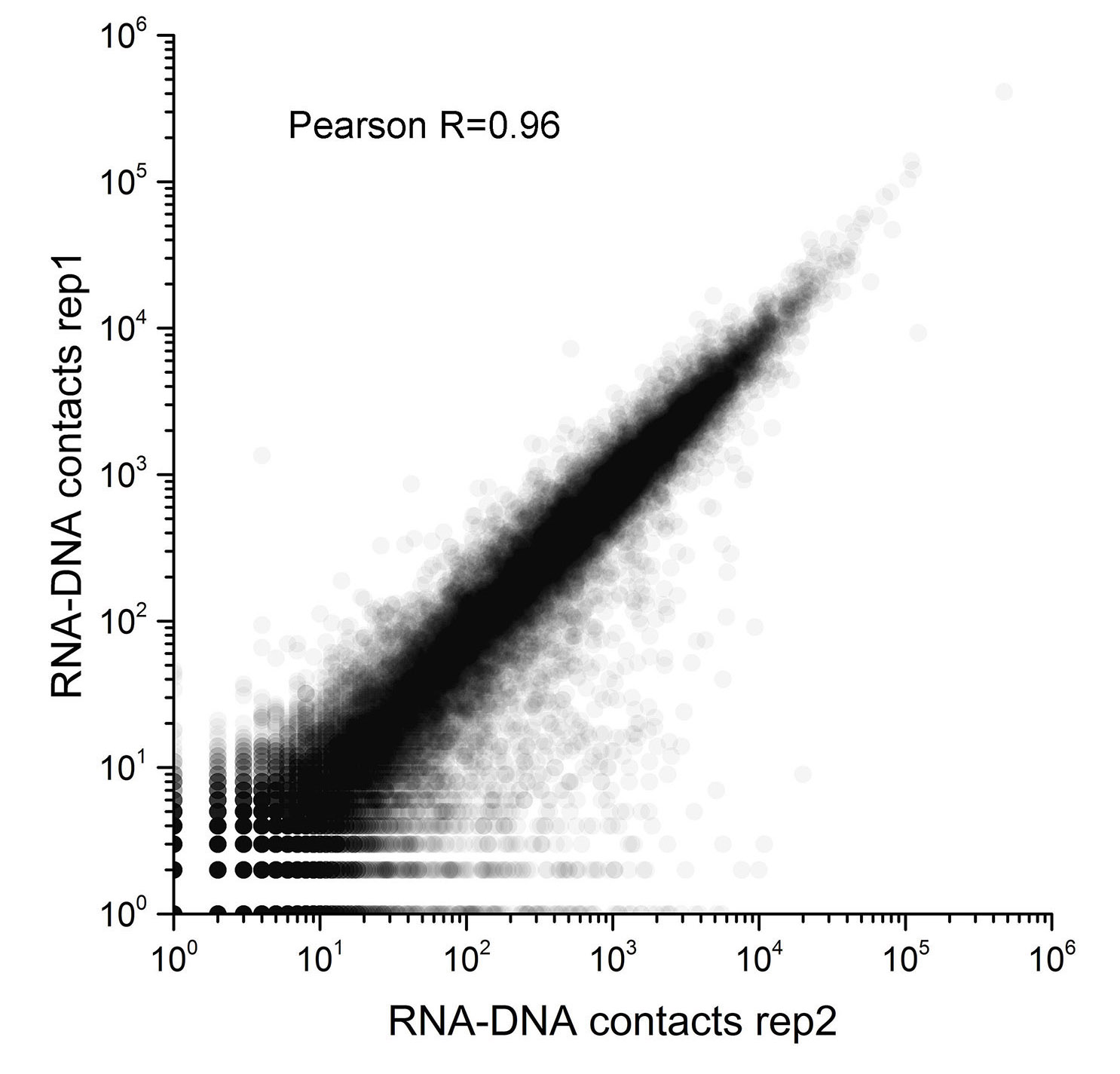

Supplement: gkaa457_Supplemental_Files [file gkaa457_supplemental_files.zip › Fig_S6.jpg]

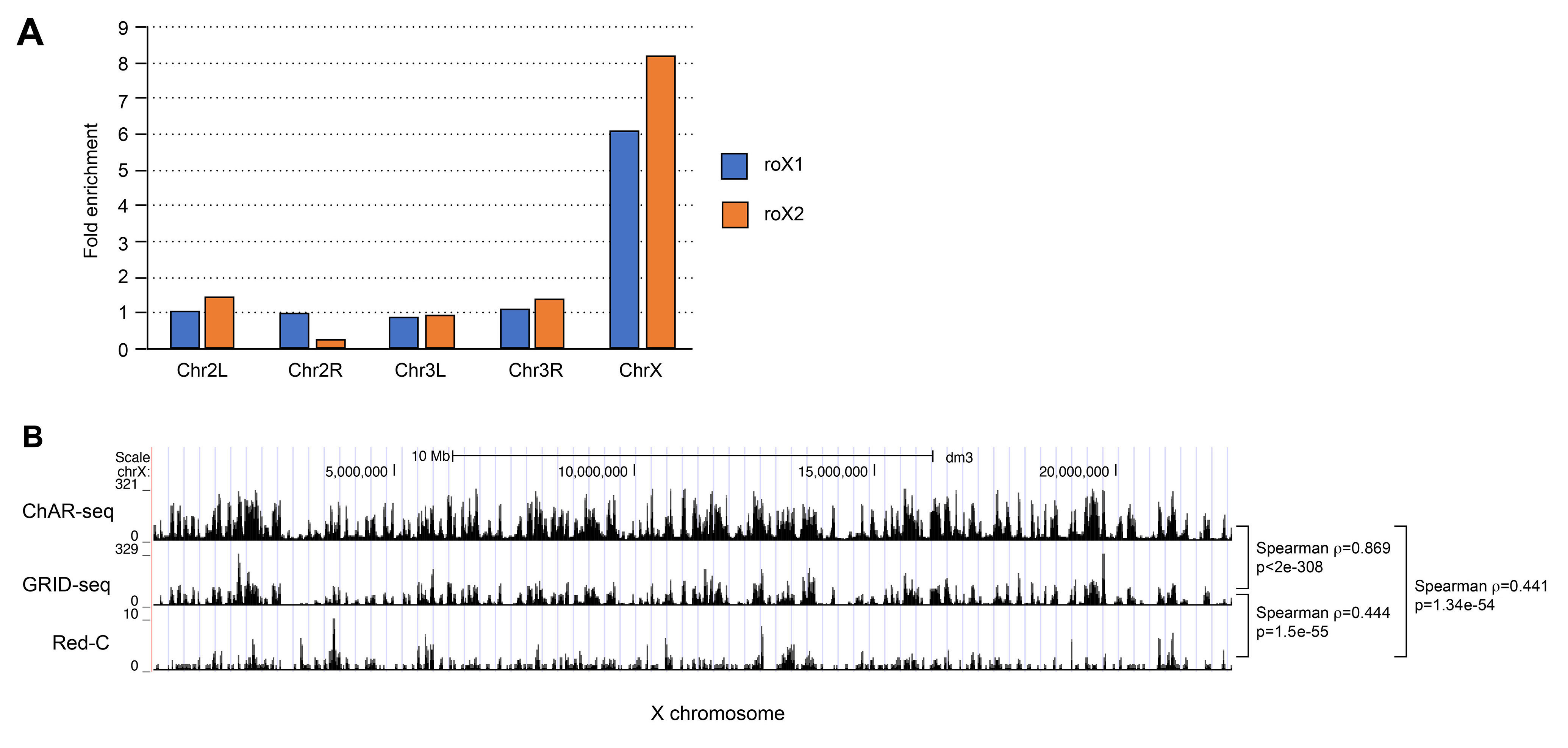

Supplement: gkaa457_Supplemental_Files [file gkaa457_supplemental_files.zip › Fig_S7.jpg]

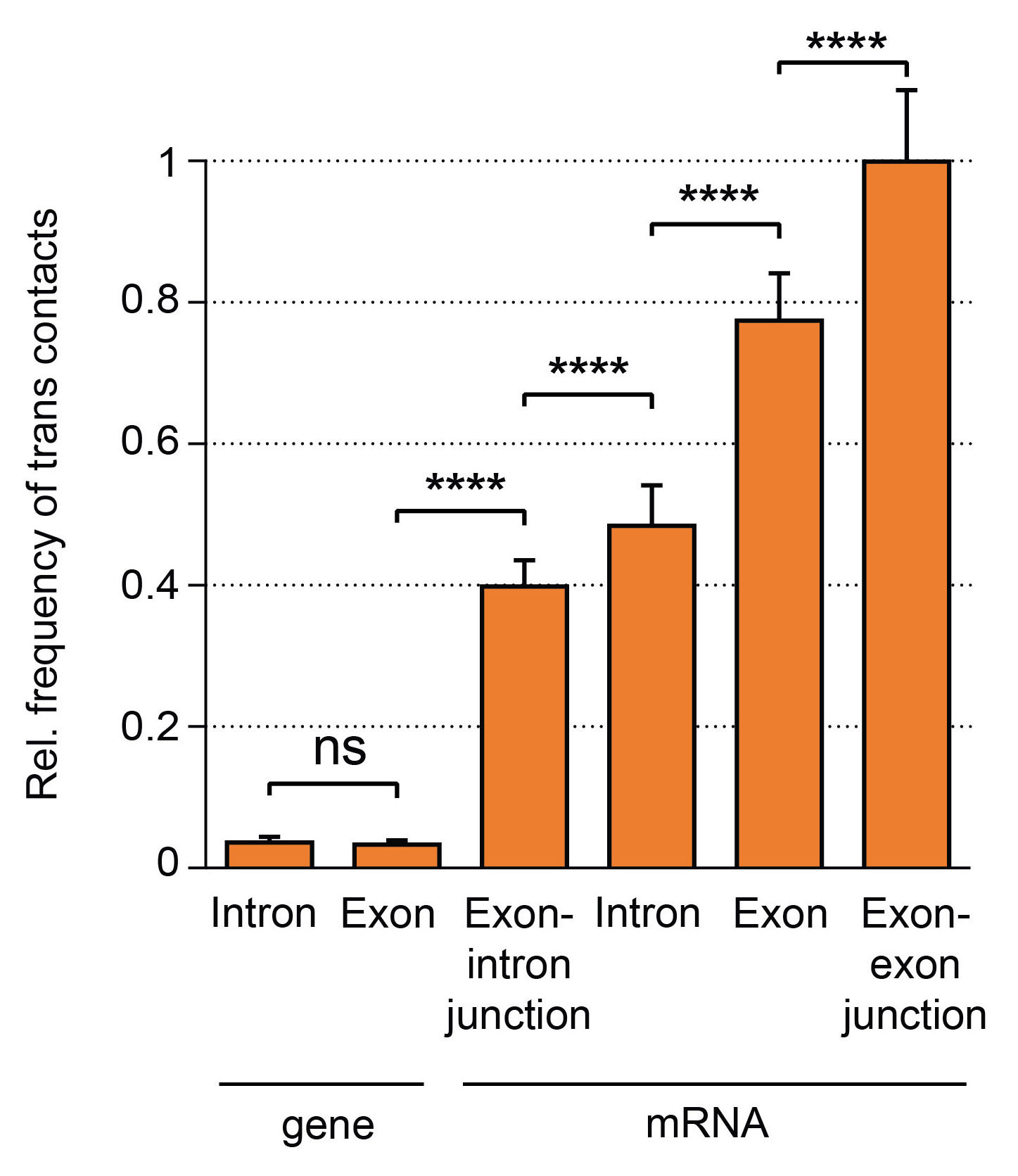

Supplement: gkaa457_Supplemental_Files [file gkaa457_supplemental_files.zip › Fig_S8.jpg]

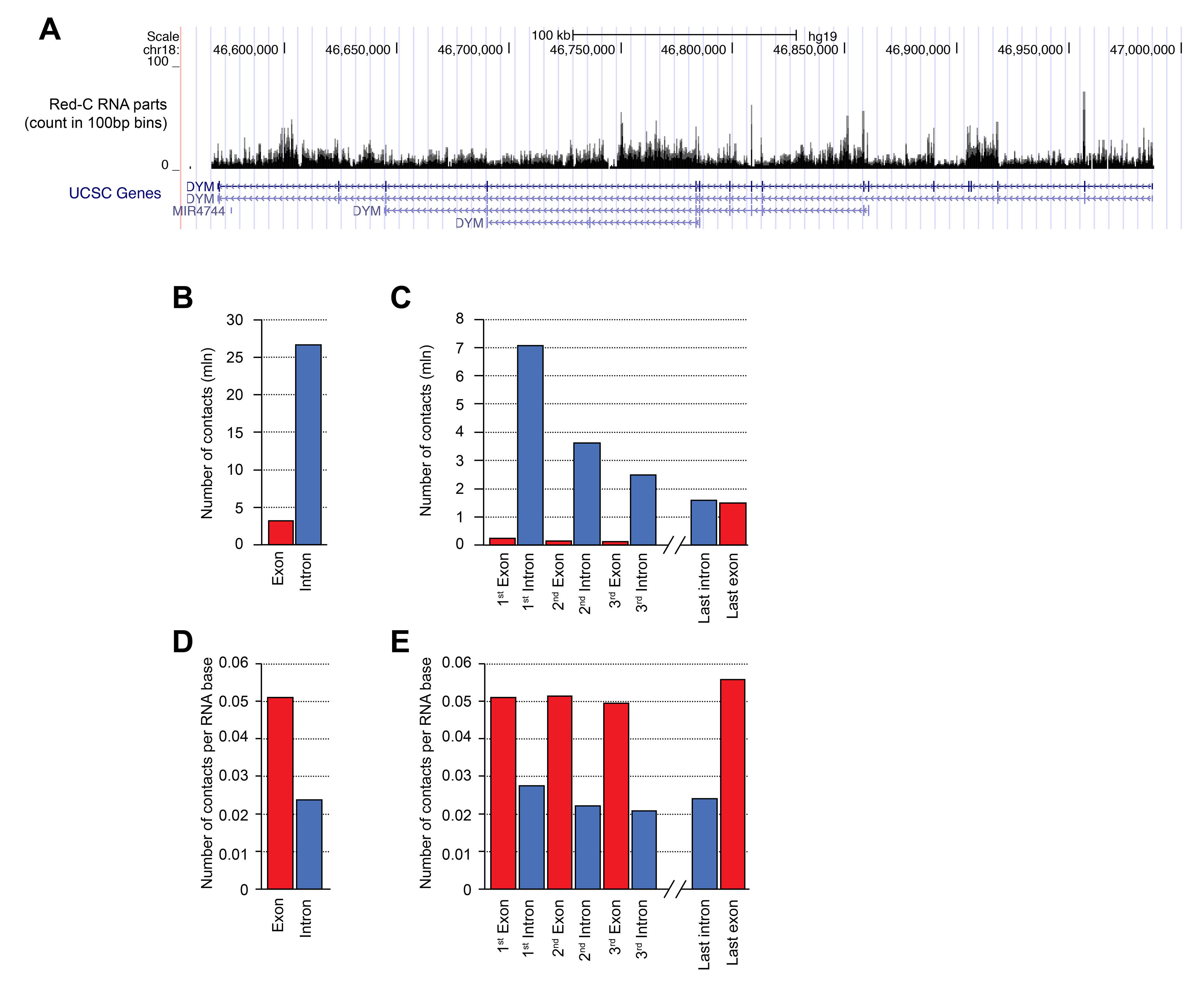

Supplement: gkaa457_Supplemental_Files [file gkaa457_supplemental_files.zip › Fig_S9.jpg]
